# Tinnitus Treatment with Trazodone

**DOI:** 10.1016/S1808-8694(15)30084-7

**Published:** 2015-10-19

**Authors:** Gabriel Cesar Dib, Cristiane Akemi Kasse, Tatiana Alves de Andrade, José Ricardo Gurgel Testa, Oswaldo Laércio M. Cruz

**Affiliations:** a M.S. MD. Otorhinolaryngologist; b PhD. MD. Otorhinolaryngologist; c MD. General Practitioner. Specialized in Geriatrics - UNIFESP-EPM; d PhD. Adjunct Professor of Otorhinolaryngology - UNIFESP-EPM; e Associate Professor - Head of the Otology Department UNIFESP-EPM. Discipline of Otorhinolaryngology and Head and Neck Surgery - Federal University of São Paulo - Paulista School of Medicine - UNIFESP-EPM

**Keywords:** neurotransmitters, serotonin, trazodone, tinnitus

## Abstract

Tinnitus is a common symptom, defined as a sound perception in absence of a sound stimulus. **Aim:** Evaluate if Trazodone, an antidepressant drug, which modulates serotonin at central neuronal pathways, is effective in controlling tinnitus. **Study Design:** Prospective, double blind, randomized, placebo-controlled. Materials and Methods: Study performed with patients presenting tinnitus. 85 patients were analyzed between February and June of 2005. 43 received trazodone and 42 placebo, for 60 days. The clinical criteria of analysis were tinnitus intensity, discomfort and life quality impact by tinnitus, using an analogue scale varying between 0 and 10, scored by patients before and after drug or placebo use. **Results:** There was a significant improvement in intensity, discomfort and life quality in both groups after treatment; however, there was no significant difference between the drug and placebo groups. Patients with age equal or over 60 years presented better results after treatment. **Conclusion:** Trazodone was not efficient in controlling tinnitus in the patients evaluated under the doses utilized.

## INTRODUCTION

Tinnitus is a frequent symptom in our clinical practice, it happens alone, or in combination with other systemic or otologic diseases. It is defined as an auditory sound perception in the absence of a generated sound stimulus, occurring from a failure in the auditory system (central and/or peripheral), encompassing the auditory sound masking^1^, ^2^.

Tinnitus is related to a number of structures of the central nervous system, and to find out the role of each of these structures is one of the major challenges associated with treatment^3^, ^4^. The most recent hypothesis used to explain its genesis and maintenance is based on the existence of a final common auditory pathway, and tinnitus would represent a failure caused by a lesion or inadequate workings at some point in this pathway, involving anatomical sites and neurotransmitters^1^, ^5^, ^6^. Auditory pathways are, in reality, a vast network of interconnected cells, from the cochlear external hair cells all the way to the temporal lobe, modulated by neurotransmitters such as dopamine, serotonin, GABA and glutamate, responsible for sound detection, location and interpretation^6^, ^7^.

Current evidences show that serotonin is able to modulate neuronal activity and the plasticity of the central pathways; therefore, altered serotonin levels in these sensorial pathways and alterations in the workings of its receptors could cause different auditory disorders, tinnitus among them. Thus, serotonin is currently seen as one of the most important neurotransmitters involved in the genesis and maintenance of this symptom^8^, ^9^.

This cellular and molecular knowledge points towards new treatment and approach possibilities insofar as tinnitus is concerned. Studies involving medication that promote proper serotonin availability in the auditory pathways could have a positive impact in the treatment of tinnitus caused by inadequate functioning of central auditory pathways, facilitating synaptic conduction and organization, as well as the modulating action within auditory inhibitory systems (efferent pathways).

Trazodone is a drug classified among a group of antidepressant medication, being considered an atypical antidepressant agent, a derivative of triazolpyridine. Its molecule does not bear association with the other drugs of similar effect, thus being different from the other drugs of this group because it has a specific chemical structure and it promotes, besides serotonin reuptake pre-synaptic inhibition (like the other medication in this group), it blocks 5-HT2A e 5-HT2C serotonin receptors in the post-neuronal synapses. 10. Thus, it bears a double mechanism of action for the increase in the serotonin levels in the central auditory pathway synapses.

Trazodone is clinically indicated for the treatment of depression, and sleep psychiatric disorders. It bears minimum anticholinergic activity, providing greater safety for the geriatric population10. The most frequent side effects are sleepiness, sedation and hypotension; and less frequently: dry mouth, gastrointestinal discomfort, dizziness, headache and hypertension. There may rarely be priapism, thus it may be used in patients with erectile dysfunction^11^, ^13^.

Throughout this medication action mechanism we could supposedly promote tinnitus symptom improvement in our patients. Thus, it seemed to us very pertinent to carry out a clinical trial to measure this possible action.

## OBJECTIVE

Our goal with the present investigation is to assess whether Trazodone acts positively in tinnitus control.

## MATERIALS AND METHODS

This is a prospective, double blind, randomized and placebo-controlled study with patients complaining of tinnitus. This research project was analyzed and approved by the Research Ethics Committee, under protocol # 1246/04.

The group of tinnitus patients added up to 104 individuals, complying with the following study inclusion criteria:

### Inclusion Criteria

• Age between 45 and 80 years.
• Both genders.
• Tinnitus with maximum progression time of one year.
• Patients with normal audiograms and mild to moderate sensorineural hearing loss.
• No defined-etiology diseases in the middle ear.
• Contra-indication regarding the use of Trazodone.
• Signature in the Informed Consent Form and Patient’s Information Letter, after being duly educated.

### Exclusion Criteria

• External and middle ear diseases or altered otoscopy.
• Conductive and mixed hearing loss.
• Severe/Profound sensorineural hearing loss.
• Use of other medication that could impact tinnitus.
• Concomitant use of other Serotonin reuptake inhibition drug or other that may act on central neurotransmitters.
• Prior use of Trazodone.

A total of 104 patients were assessed between February and June of 2005. Of the 85 who remained in the study until the end, 43 patients received the medication and 42 received placebo.

Prior to the use of the drug or placebo, we carried out tonal and vocal audiometry and impedanciometry. The medication was used in the dose of 50mg per tablet, through a single night dose for 60 continuous days. If important side effects were seen, the medication was discontinued.

Clinical analysis criteria were intensity, life quality impact and level of discomfort caused by tinnitus, by means of using an analogue scale with scores from 0 to 10 given by the patient before and after using trazodone or placebo. We compared the relationship between audiogram profile, gender, age equal to or above 60 years and intensity level before treatment and the analysis criteria.

We assessed whether tinnitus intensity levels higher or lower before treatment had any association with tinnitus intensity after treatment. For that we created the intensity difference variable (final intensity minus the initial intensity). We then calculated the Pearson’s correlation coefficient with the pre-treatment intensity and we could then check to see if the higher or lower difference had any association with the higher or lower tinnitus intensity before treatment.

The tablets (drug and placebo) were similar in shape, color and size, identified only by the numbers “23” and “24” by the pharmaceutical company that provided the medication. Only the pharmacist knew what drug was being given to which patient until the end of drug use and the clinical evaluation of the patients.

At the end of the study we learned that medication number “23” was the active drug (Trazodone) and the number “24” was the placebo.

We used the ANOVA (variance analysis), chi-squared and t-independent statistical methodologies to analyze the data obtained. 5% was the test significance level, and the data were deemed significant when p was less than or equal to 0.05.

## RESULTS

The groups were homogeneous in gender, tinnitus dwelling time, form of onset, frequency and audiometric profile.

The results obtained and the group of patients evaluated in our study is presented in tables. We evaluated 25 women (58.1%) and 18 men (41.9%) who used Trazodone; and 31 women (73.8%) and 11 men (26.2%) with placebo.

As to how frequent the patients had tinnitus, we assessed 36 patients (83.7%) with continuous tinnitus and 7 with intermittent tinnitus who used trazodone, and 37 patients (88.1%) with continuous tinnitus, 5 intermittent (11.9%) used placebo.

The groups were homogeneous (p=0.69). In both groups there was improvement after medication use (p<0.0001); however, there was no statistically significant difference between the drug and placebo groups (p=0.59).

**Table 1:** Tinnitus dwelling time in years for the patients evaluated in this study.

	Drug	Placebo
Mean value	6.07	6.55
Standard deviation	5.05	6.83
N	43	42

Test t (p) = 0.715

**Table 2:** Tinnitus onset in the patients evaluated in this study.

	Drug	Placebo		
	N	%	N	%
Gradual	33	76.7	31	73.8
Sudden	10	23.3	11	26.2

Total	43	100.0	42	100.0

Chi-squared test (p) = 0.754

**Table 3:** Audiometric profile of the patients evaluated in this study.

	GROUP				Total	
	Drug	Placebo				
	N	%	N	%	N	%
Mild	17	39,5%	16	38%	33	38,8%
Moderate	11	25,5%	14	33,4%	25	29,4%
Normal	15	35%	12	28,6%	27	31,8%

Total	43	100,0%	42	100,0%	85	100,0%

**Table 4:** Tinnitus intensity as reported by the patients before and after treatment - mean values, standard deviations and variance analysis.

		Initial	Post
	Mean value	6,60	5,86
Drug	Standard deviation	1,59	2,33
	N	43	43
	Mean value	6,55	5,62
Placebo	Standard deviation	1,60	2,08
	N	42	42

The groups were homogeneous (p=0.10). In both groups there was improvement after medication use (p<0.0001); however, there was no statistically significant difference between the drug and placebo groups (p=0.41). There was an indication that the drug was associated with greater discomfort.

The groups were homogeneous (p=0.26). In both groups there was an improvement after medication use (p<0.0001); however, there was no statistical difference between the drug and placebo groups (p=0.77).

**Table 5:** Level of discomfort caused by the tinnitus before and after treatment - mean values, standard deviations and variance analysis.

		Initial	Post
	Mean value	6,56	5,91
Drug	Standard deviation	1,86	2,50
	N	43	43
	Mean value	6,02	5,10
Placebo	Standard deviation	1,70	1,96
	N	42	42

**Table 6:** Tinnitus impact on the patients’ life quality before and after treatment - mean values, standard deviations and variance analysis.

		Initial	Post
	Mean value	6,12	5,49
Drug	Standard deviation	1,82	2,39
	N	43	43
	Mean value	5,71	5,00
Placebo	Standard deviation	1,66	1,96
	N	42	42

**Table 7:** Tinnitus intensity reported by the patients before and after treatment according to their hearing levels - mean values, standard deviations and variance analysis.

		Normal		Mild		Moderate	
		Initial	Post	Initial	Post	Initial	Post
	Mean value	6,87	5,93	6,82	6,12	5,91	5,36
Drug	Standard deviation	1,46	2,69	1,55	1,87	1,76	2,58
	N	15	15	17	17	11	11
	Mean value	6,58	6,08	6,44	5,50	6,64	5,36
Placebo	Standard deviation	1,38	1,24	1,71	2,28	1,74	2,47
	N	12	12	16	16	14	14

The groups were homogeneous (p=0.86). Both groups improved after medication (p<0.0001); however, there was no statistical difference between the drug and placebo groups (p=0.41).

The groups were homogeneous (p=0.14). In both groups there was an improvement after the use of medication (p<0.0001); however, there were no statistical difference between the drug and placebo groups (p=0.31).

The groups were homogeneous (p=0.35). In both groups there was improvement after medication use (p<0.0001); however, there was no statistical difference between drug and placebo (p=0.58).

Therefore, there was no relation between the audiometric profile and intensity, discomfort and life quality impact before and after treatment.

The groups were homogeneous (p=0.82). In both groups there was an improvement after the use of medication (p<0.0001); however, there was no statistical difference between the placebo and drug groups (p=0.72).

The groups were homogeneous (p=0.82). In both groups there was an improvement after medication use (p<0.0001); however, there were no statistically significant differences between the drug and placebo groups (p=0.72).

The groups were homogeneous (p=0.34). In both groups there was improvement after medication use (p<0.0001); however, there was no statistically significant difference between the drug and placebo groups (p=0.73).

**Table 8:** Level of discomfort caused by tinnitus reported by the patients before and after treatment, according with their hearing levels - mean values, standard deviations and variance analysis.

		Normal		Mild		Moderate	
		Initial	Post	Initial	Post	Initial	Post
	Mean value	6,60	5,80	6,82	6,18	6,09	5,64
Drug	Standard deviation	1,64	2,65	1,59	1,88	2,51	3,23
	N	15	15	17	17	11	11
	Mean value	5,92	5,58	6,25	4,88	5,86	4,93
Placebo	Standard deviation	1,68	1,51	1,57	2,16	1,96	2,13
	N	12	12	16	16	14	14

**Table 9:** Tinnitus impact on the patient’s life quality before and after treatment according to hearing levels - mean values, standard deviations and variance analysis.

		Normal		Mild		Moderate	
		Initial	Post	Initial	Post	Initial	Post
	Mean value	6,20	5,60	6,41	5,65	5,55	5,09
Drug	Standard deviation	1,57	2,38	1,66	2,06	2,34	3,02
	N	15	15	17	17	11	11
	Mean value	5,67	5,42	5,88	5,00	5,57	4,64
Placebo	Standard deviation	1,37	1,31	1,63	2,07	1,99	2,34
	N	12	12	16	16	14	14

**Table 10:** Tinnitus intensity reported by the patients before and after treatment, broken down by gender, in terms of mean values, standard deviations and variance analysis.

		Drug		Placebo	
		Initial	Final	Initial	Final
	Mean value	6,84	6,04	6,58	5,55
Females	Standard deviation	1,60	2,35	1,57	2,10
	N	25	25	31	31
	Mean value	6,28	5,61	6,45	5,82
Males	Standard deviation	1,56	2,33	1,75	2,14
	N	18	18	11	11

**Table 11:** Tinnitus-caused level of discomfort reported by the patients before and after treatment, according to gender - mean values, standard deviations and variance analysis.

		Drug		Placebo	
		Initial	Final	Initial	Final
	Mean value	6,76	6,08	6,13	5,06
Females	Standard deviation	1,74	2,45	1,78	2,03
	N	25	25	31	31
	Mean value	6,28	5,67	5,73	5,18
Males	Standard deviation	2,02	2,61	1,49	1,83
	N	18	18	11	11

**Table 12:** Life quality impact of tinnitus before and after treatment broken down by gender - mean values, standard deviations and variance analysis.

		Drug		Placebo	
		Initial	Final	Initial	Final
	Mean value	6,36	5,68	5,77	4,97
Females	Standard deviation	1,66	2,36	1,65	1,94
	N	25	25	31	31
	Mean value	5,78	5,22	5,55	5,09
Males	Standard deviation	2,02	2,49	1,75	2,12
	N	18	18	11	11

**Table 13:** Association between age equal to or less then 60 years and tinnitus intensity before and after treatment - mean values, standard deviations and variance analysis.

		Drug		Placebo	
		Initial	Final	Initial	Final
	Mean value	7,00	6,35	7,00	6,86
< 60 years	Standard deviation	1,41	2,23	1,24	1,41
	N	26	26	14	14
	Mean value	6,00	5,12	6,32	5,00
>= 60 years	Standard deviation	1,70	2,34	1,72	2,11
	N	17	17	28	28

**Table 14:** Association between age equal to or higher than 60 years and tinnitus-related discomfort reported by the patients before and after treatment - mean values, standard deviations and variance analysis.

		Drug		Placebo	
		Initial	Final	Initial	Final
	Mean value	6,88	6,27	6,29	6,07
< 60 years	Standard deviation	1,58	2,25	1,14	1,33
	N	26	26	14	14
	Mean value	6,06	5,35	5,89	4,61
>= 60 years	Standard deviation	2,16	2,80	1,93	2,06
	N	17	17	28	28

Therefore, there was no association between gender and the responses regarding intensity, discomfort and life quality impact before and after treatment.

We assessed if the higher or lower tinnitus intensity levels before treatment had any association with the tinnitus intensity improvement after treatment, and we obtained the Pearson’s correlation index of -0.024 (p=0.825).

patients when we compared trazodone and placebo.

There were no statistically significant differences in relation to hearing loss being present or not, and in both

There was no significance and, therefore, no relation between the tinnitus initial intensity level and after-treatment improvement.

The groups were homogeneous (p=0.63). In both groups there was improvement after treatment (p<0.0001), and the group with age equal to or higher then 60 years presented with less intensity tinnitus after treatment (p=0.04).

The groups were homogeneous (p=0.30). In both groups there was improvement after treatment (p<0.0001), and the group with age above or equal to 60 years presented less tinnitus-related discomfort after treatment (p=0.09).

The groups were homogeneous (p=0.68). In both groups there was improvement after treatment (p<0.0001), and the group with age equal to or higher then 60 years presented the least tinnitus-related impact on life quality after treatment (p=0.08).

The age range of age equal to or higher than 60 years presented less values of intensity, discomfort and life quality impact after treatment. There was a greater reduction of such factors in the placebo group.

The statistical test was not used because of the low incidence of side effects presented by the patients.

The statistical test was not used because of the low incidence of side effects presented by the patients.

**Table 15:** Association between age equal to or higher then 60 years and the tinnitus-caused impact on life quality reported by the patients before and after treatment - mean values, standard deviations and variance analysis.

		Drug		Placebo	
		Initial	Final	Initial	Final
	Mean value	6,58	6,00	6,00	5,93
< 60 years	Standard deviation	1,63	2,24	1,24	1,33
	N	26	26	14	14
	Mean value	5,41	4,71	5,57	4,54
>= 60 years	Standard deviation	1,91	2,47	1,83	2,08
	N	17	17	28	28

**Table 16:** Adverse effects the patients in the study presented.

	GROUP				Total	
	D		P			
	N	%	N	%	N	%
No adverse effects	36	83,7%	39	92,9%	75	88,2%
Apathy	1	2,3%	0	0,0%	1	1,2%
Sour mouth	0	0,0%	1	2,4%	1	1,2%
Hypertensive crisis	1	2,3%	0	0,0%	1	1,2%
Epigastralgia	1	2,3%	0	0,0%	1	1,2%
Insomnia	0	0,0%	1	2,4%	1	1,2%
Nausea	1	2,3%	0	0,0%	1	1,2%
Sleepiness	3	7,0%	1	2,4%	4	4,7%

Total	43	100,0%	42	100,0%	85	100,0%

The statistical test was not used because of the low incidence of side effects presented by the patients.

**Table 17:** Other improvements reported by the patients who participated in the study.

	GROUP				Total	
	Drug		Placebo			
	N	%	N	%	N	%
Not reported	35	81,4%	40	95,2%	75	88,2%
Irritability reduction	2	4,7%	0	0,0%	2	2,4%
Improve in sleep	6	14,0%	2	4,8%	8	9,4%

Total	43	100,0%	42	100,0%	85	100,0%

The statistical test was not used because of the low incidence of side effects presented by the patients.

## DISCUSSION

Where tinnitus is concerned, there are a number of neurotransmitters acting in the central pathways and interacting among themselves, it may be for this reason that we do not find positive results acting in the modulation of one neurotransmitter only, and also for the fact that there may be other anatomical sites involved in the genesis and maintenance of tinnitus^8^, ^9^, and the lack of proven efficient methods to detect where in the central auditory pathway is the tinnitus triggering point^3^, ^4^, ^14^. It is very likely that in order to control this symptom, we will need to develop drugs that act on the many anatomical sites and also methods to locate the specific site responsible for the genesis and maintenance of tinnitus in the central auditory pathways.

Another very important fact is the need to approach these patients in the acute phase (early on) of tinnitus onset, before it centralizes, preventing plastic changes to occur in the central nervous system, causing tinnitus chronification8. This explains why, in the acute phase, tinnitus is more easily treated and with better clinical outcome. When it becomes chronic, the patient loses the habituation mechanism^15^ and, consequently, treatment becomes complex because of central alterations.

Psychological and psychiatric factors are often times present in tinnitus patients, that triggers or accruing from it, are greatly relevant, and this alone would justify treatment with antidepressant agents^16^, ^17^, ^18^. Thus, satisfactory results corroborate a double mechanism for tinnitus improvement in these patients, in other words, acting on the neurotransmitters of the central auditory pathway receptors and on psychogenic/depression factors^19^, ^20^, ^21^. There are reports of tinnitus onset or return in patients under use of antidepressant medication when it was discontinued because of improvements in the depression^22^, ^23^.

In our study we did not find significant improvements in tinnitus intensity and life quality among these cases there was not improvement in tinnitus intensity, discomfort and life quality before and after the use of these agents.

This tells us that the patient having or not having hearing loss did not have any positive influence in tinnitus improvement with the use of trazodone.

Comparing gender and improvement before and after the use of the drug or placebo, we did not find any significance. We then inferred that gender, despite the fact that tinnitus is more prevalent in females, does not represent a predictive factor for improvement.

Prior tinnitus intensity did not contribute to treatment improvement, as shown by the Pearson’s correlation coefficient, probably because of tinnitus centralization and chronification, regardless of level of intensity.

When we analyzed the relationship between patients with ages equal to or above 60 years and tinnitus intensity improvement, discomfort and life quality impact before and after treatment, we noticed significance for them in this age range, with a greater reduction in these factors, particularly in the placebo group. This may indicate that in elderly patients, antidepressant medication may improve tinnitus, especially in the placebo group. This may indicate that in the elderly patients, antidepressants may improve tinnitus, especially because depressive symptoms are more frequent in this age range, and also because of the good doctor-patient relationship, which could also explain a greater improvement in the patients of the placebo group, by giving the patient attention, advice, clearing up doubts and answering the patient’s questions regarding their tinnitus.

It was interesting to hear some patients complaining of sleepiness after using trazodone, reported as a desirable effect, because they started to sleep better, having less sensation of tinnitus at night, when usually the patients complain more of this symptom. Other patients reported it as an undesirable effect, because it affected their daily activities11. There was a small incidence of important adverse effects, and it was necessary to discontinue the treatment in one patient, who had a hypertension crisis on the third day of trazodone, very likely medication-related.

The initial dose used was 50 to 150mg/day, divided in two doses or in a single daily dose taken at night^10^, ^11^, ^12^. It may be that by using higher trazodone doses (100 to 150 mg/day), as the doses used to treat depression, more satisfactory results could be attained, since tinnitus happens because of alterations in the activities of neurotransmitters and their receptors. Thus, there would be a more pronounced improvement of this symptom due to a greater availability of serotonin in the central auditory pathways, because of a greater block of its post-synaptic receptors^12^, ^24^.

This study was important in order to identify that, low medication doses that act on serotonin modulation in the central auditory pathway synapses may not be efficient in improving clinical signs and symptoms, despite minimum side effects. Prolonged use, in higher doses, could improve even further the tinnitus discomfort; however, severe side effects and medication addiction are relevant facts that should be considered.

Further studies on the central auditory pathways’ neurotransmitters are necessary in order to better explain the role of each one in the genesis and maintenance of tinnitus, so that we may provide our patients with a better therapeutic approach.

## CONCLUSION

In the dose used, trazodone was not efficient in controlling tinnitus for our group of patients.
